# Beyond the Common Femoral Artery: The Overlooked Risk of External Pudendal Artery Injury

**DOI:** 10.1155/cric/9570668

**Published:** 2026-05-14

**Authors:** Ayham Al-Shatanawi, Uzma Aftab, Patrick Tran, Sohail Q. Khan

**Affiliations:** ^1^ Department of Cardiology, University Hospitals Birmingham NHS Foundation Trust, Birmingham, UK, nhs.uk; ^2^ Faculty of Medicine, The Hashemite University, Zarqa, Jordan, hu.edu.jo; ^3^ Centre for Health and Life Sciences, Coventry University, Coventry, UK, coventry.ac.uk; ^4^ Department of Cardiology, University Hospitals Coventry & Warwickshire, Coventry, UK, nhs.uk; ^5^ Institute of Cardiovascular Science, University Hospitals Birmingham NHS Foundation Trust, Birmingham, UK, nhs.uk

**Keywords:** coil embolisation, external pudendal artery, femoral access complication, percutaneous coronary intervention

## Abstract

Femoral arterial access is a cornerstone of numerous interventional procedures including percutaneous coronary intervention (PCI), structural valve and neurovascular interventions. Although common femoral artery (CFA) injuries are well‐recognised, branch‐vessel injuries, such as the external pudendal artery, remain underreported, despite the potential for significant bleeding and haemodynamic compromise. We present a 91‐year‐old lady who developed a rapidly expanding groin haematoma following PCI via femoral access, despite optimal technique using a micropuncture access kit under ultrasound guidance. She developed haemodynamic collapse, necessitating multiple blood transfusions. CT angiography revealed active extravasation from the external pudendal artery, which was successfully managed with coil embolisation. The patient stabilised postprocedure and was eventually discharged home. As PCI expands to older and higher risk cohorts, this case underscores the need for heightened awareness of branch vessel anatomy, advocating for a multimodal approach integrating fluoroscopic wire tracking with ultrasound guidance to mitigate branch vessel injury.

## 1. Introduction

Although the radial artery approach has gained preference in many cardiovascular procedures due to lower complication rates, femoral arterial access remains critical in certain interventions including complex percutaneous coronary interventions (PCIs), structural valvular and neurovascular interventions. Despite advancements in access‐site management techniques, femoral artery complications persist including pseudoaneurysm, haematoma and external and retroperitoneal bleeding in > 10% of cases, depending on the patient and procedural risk factors [1, 2].

Although common femoral artery (CFA) injuries are well‐recognised, the involvement of smaller branch vessels, such as the superficial circumflex iliac artery and external pudendal artery (EPA) is less frequently reported but can lead to significant morbidity [3]. Injury to these branches may not be immediately apparent and can result in progressive bleeding requiring urgent intervention. Here, we present a rare case of post‐PCI groin haematoma expanding into the vulva arising from injury to a branch of the EPA, despite the use of a micropuncture access kit under ultrasound guidance. This case highlights the challenges of femoral access across interventional fields and the need for heightened awareness of this vascular complication to enhance patient safety.

## 2. Case Presentation

### 2.1. History of Presenting Complaint

This 91‐year‐old lady, who is normally independent with preserved cognitive function, presented to the emergency department with sudden onset excruciating epigastric pain which radiated to the chest and upper back. It started following her evening meal and continued throughout the night, and was associated with mild exertional breathlessness the following morning. There was no associated diaphoresis or nausea. She denied cough, palpitations or feeling lightheaded. Her past medical history included only a hiatus hernia and benign gastric tumour. She was not on any regular medications and was allergic to cefalexin and nitrofurantoin.

### 2.2. Examination

Initial examination revealed a raised blood pressure (161/83 mmHg) and normal heart rate (85/min), resting oxygen saturation 98% on room air and temperature 35.9°C. Her body mass index was 19.53. Apart from mild bilateral ankle pitting oedema, cardiovascular examination was unremarkable. Abdomen was soft and nontender, specifically no epigastric tenderness on deep palpation. Chest auscultation revealed equal bilateral air entry.

### 2.3. Initial Workup

Initial electrocardiography (ECG) showed T wave inversions in leads V2–V4 with no ST segment elevations or depressions. Serial ECGs obtained every 30 min showed no dynamic changes. Laboratory findings revealed elevation in Troponin I and N‐terminal pro‐B type natriuretic peptide (NT‐proBNP) at 2679 and 3941 ng/L, respectively, confirming significant myocardial injury and strain. Age‐adjusted D‐dimer was within normal limits at 910 ng/mL. Other blood investigations included platelets 132 × x10^9^/L, haemoglobin 132 g/L with normal liver function tests and an estimated glomerular filtration rate of 72 mL/min/1.73m^2^. INR was 1.2. CT angiogram aorta excluded acute aortic syndrome but showed severe triple vessel coronary artery calcification and subendocardial hypoattenuation within multiple LV segments in the left anterior descending artery (LAD) territory, consistent with a myocardial infarction. Transthoracic echocardiogram revealed a normal‐sized left ventricle with borderline reduced systolic function, with a left ventricular ejection fraction of 53% (± 5%) by Simpson′s biplane method. Diastolic dysfunction with elevated filling pressures was present. No haemodynamically significant valvular heart disease was detected.

Patient was managed as having a non‐ST segment elevation myocardial infarction (NSTEMI) with dual antiplatelet therapy (aspirin 75 mg once daily and ticagrelor 90 mg twice a day following a loading dose), low‐molecular‐weight heparin, atorvastatin 80 mg and bisoprolol 2.5 mg once daily. Following discussion of the benefits and risks of coronary angiography ± PCI, the patient elected for invasive management.

### 2.4. PCI

**Figure 1:**
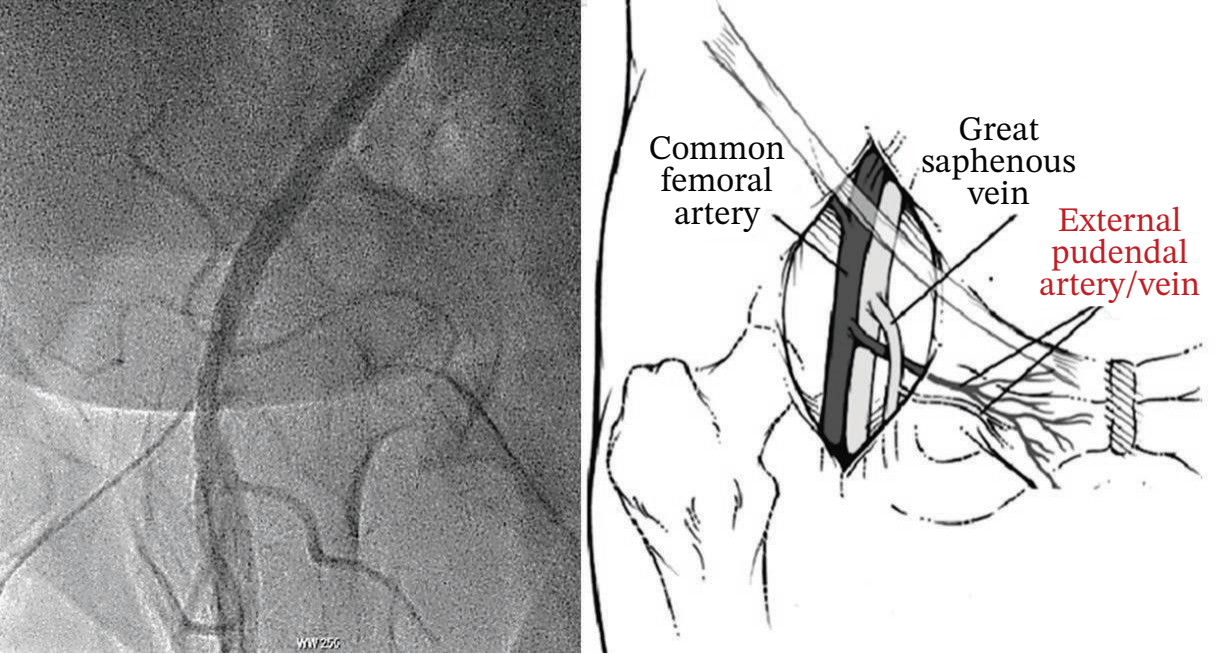
(Right) Straight left anterior oblique view confirmed that the micropuncture sheath was in appropriate position (above femoral bifurcation and below inferior epigastric artery); (left) external pudendal artery arises medially from the proximal common femoral artery and crosses anteriorly over the common femoral vein.

Initial ultrasound scan revealed small calibre right radial and ulnar arteries, prompting transition to a femoral approach. The right femoral arterial access was obtained on the first attempt using a Micropuncture introducer kit (Galt Medical Corp., Garland, Texas, United States). Real‐time ultrasound demonstrated the needle tip in the CFA lumen, midway between the inguinal ligament and bifurcation. Fluoroscopy confirmed the position (Figure 1). A 0.018‐inch guidewire advanced smoothly without resistance and the 4‐Fr sheath was introduced without complication. Transition to a 6‐Fr femoral sheath proceeded uneventfully. In this case, the femoral vein was neither used for venous sampling nor for central venous catheterization.

Coronary angiography revealed moderate to severe atherosclerotic disease of the left main stem (LMS). The LAD had severe ostial disease with significant calcification, whereas moderate disease was present in the mid and distal segments. Moderate disease was present in both the left circumflex and right coronary arteries.

Intravascular ultrasound (IVUS) of the LAD confirmed the presence of heavy calcification. Given the severity of the disease, PCI was performed on both the LMS and LAD using noncompliant balloons before deploying one drug‐eluting stent (BioFreedom). Throughout the procedure, she remained haemodynamically stable. A total of 6000 IU of heparin was administered, and the activated clotting time was 253 s without using glycoprotein IIb/IIIa inhibitors. Following PCI, manual compression was applied to the right groin puncture site, where a small haematoma was noted; this was managed using a SafeGuard Pressure–assisted Device to maintain consistent pressure over this site.

### 2.5. Postprocedure

The patient developed dizziness in the recovery room, accompanied by hypotension with a blood pressure of 66/50 mmHg and bradycardia at 52 bpm. Clinical examination revealed progression of the haematoma in the right groin. ECG and echocardiography were performed, which showed no evidence of ischaemia or pericardial effusion, respectively. However, the haemoglobin level dropped significantly to 70 g/L, prompting the need for fluid resuscitation and blood transfusion. In an attempt to achieve haemostasis, a FemoStop Compression System Device was applied for 6 h. Despite this, the haematoma rapidly expanded medially, resulting in marked swelling of the labia majora and mons pubis.

**Figure 2:**
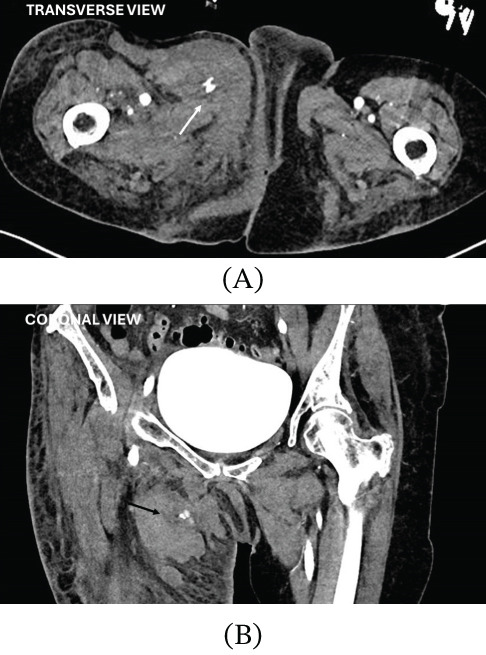
CT angiography demonstrating an extensive right groin haematoma with active contrast extravasation. (A) Transverse view: White arrow indicates active contrast extravasation within the haematoma, which extends medially along fascial planes towards the perineum. (B) Coronal view: Black arrow demonstrates a large haematoma (approximately 8 × 7 cm) within the right femoral triangle, with medial tracking into the vulval region.

**Figure 3:**
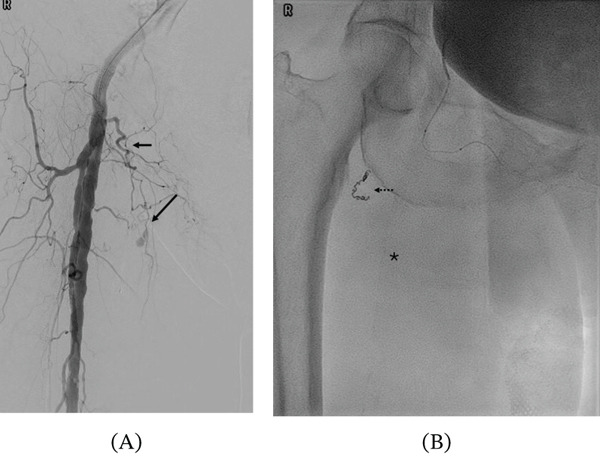
Selective angiography of the external pudendal artery demonstrating active bleeding and subsequent embolisation. (A) Angiogram showing the external pudendal artery (solid arrow) with active contrast extravasation from distal branches. (B) Postembolisation image demonstrating successful occlusion following deployment of 2‐mm Nester coils (dashed arrow). Asterisk indicates the treated distal vascular territory; the glue cast is not clearly visualised due to the small volume used and dilution (1:4) with ethiodized oil.

Bedside doppler ultrasonography demonstrated a pulsatile superficial femoral artery and CFA with a large haematoma and thrombosed blood medially. No arteriovenous fistula or pseudoaneurysm was noted. A CT angiogram of the aorta revealed a large haematoma (8 × 7 cm) in the right femoral triangle with features suggestive of active bleeding from a branch of right EPA (Figure 2). Interventional radiology was consulted, and an arterial embolisation was performed through left CFA using Cobra catheter (Cook Medical, United States) with delivery of Progreat microcatheter (Terumo, Japan) to deliver 2‐mm Nester (Cook Medical, United States) coils targeting the branch of EPA. Postembolisation angiography revealed continued bleeding from a distal branch of the EPA, which was successfully embolised using a small volume of Glubran (GEM Srl, Italy) synthetic glue (1:4 dilution) with Lipiodol Ultra Fluid (ethiodized oil) (Figure 3). Given the dilution and small volume used, it was not visible on imaging.

### 2.6. Follow‐Up

The patient was closely monitored following the intervention. Antiplatelet therapy was cautiously resumed, and over the subsequent 2 days, haemoglobin remained stable and the haematoma gradually reduced in size. She was mobilising at baseline and discharged home.

## 3. Discussion

Femoral access for coronary, structural and neuro‐interventions continues to carry a higher risk of local vascular complications—including haematomas, pseudoaneurysm and arteriovenous fistulas—compared with the transradial approach [4]. Among these, bleeding from small arterial branches such as the EPA remains exceptionally rare, particularly when best practices are followed, as in the case presented here.

### 3.1. Minimising Risks During Femoral Arterial Puncture

Optimal femoral artery cannulation should occur at the level of the CFA, above its bifurcation and below the origin of the inferior epigastric artery. This corresponds to approximately 2–3 cm below the midpoint between the anterior superior iliac spine and pubic symphysis [5]. Puncturing at the midfemoral head where the femoral artery is supported by the femoral head allows manual compression for haemostasis. The most critical operator‐related factor in minimising iatrogenic bleeding is accurate puncture location. This risk can be significantly reduced with a combination of real‐time ultrasound‐guided puncture, use of Micropuncture kits and limiting femoral sheath size to 6 Fr or smaller [6]. [6] demonstrated that ultrasound‐guided femoral puncture substantially lowered the incidence of major bleeding in patients receiving arterial closure device [7]. Compared with manual compression, vascular closure devices have a better outcome in achieving haemostasis after PCI [8]. Despite adhering to these best practices, our patient developed an expanding groin haematoma, underscoring that even with ideal technique, rare but serious vascular complications can still arise, especially in high‐risk individuals.

### 3.2. Anatomical and Patient‐Specific Considerations

Patient‐related risk factors for haematoma development after femoral access PCI include advanced age, female gender, extremes of body mass index, multiple comorbidities including impaired renal function, low platelet count and peripheral vascular disease and anticoagulation before and after PCI and higher than usual bifurcation of femoral artery into main branches [9]. Our patient had many of these adverse factors which could have contributed to haematoma development despite the maximal caution by the operator. Anatomically, the EPA arises medially from the CFA, just distal to the inguinal ligament and courses horizontally and superficially towards the perineum, crossing anterior to the femoral vein, making it vulnerable to injury during common femoral vein cannulation and potentially leading to arteriovenous fistula or bleeding complications. The anatomy of the EPA can also vary, with some individuals having both superficial and deep branches. Injury to the EPA can occur if the femoral arterial puncture is too low or medial, especially in cases of high bifurcating CFA, resulting in delayed or active bleeding, as seen here.

### 3.3. Current Literature

Reports of external pudendal artery injury during femoral access are rare in the current literature. Lønnebakken et al. reported iatrogenic injury of the EPA resulting in a pseudoaneurysm and communicating arteriovenous fistula, following cardiac catheterisation [10]. Similarly, Algin et al. described a pseudoaneurysm of the superficial EPA during CFA puncture for coronary angiography [11]. The absence of Doppler ultrasound guidance for arterial puncture, coupled with anticoagulation and use of a large sheath, was thought to have contributed to these cases. Otherwise, the majority of reported cases of EPA injury relate to genital tract trauma and orthopaedic interventions [12, 13].

In our case, both ultrasound and fluoroscopic guidance were used for CFA access. One potential mechanism of branch vessel injury away from the puncture site could be inadvertent guidewire advancement, particularly with micropuncture guidewires, which, although providing a degree of tactile feedback, have a greater tendency to track into small branch vessels due to their flexibility and can unintentionally enter smaller branches like the EPA, leading to wire perforation. This risk can be minimised by fluoroscopic guidance to visualise the wire while advancing it into the lumen of the desired vessel. This case highlights the need to employ a technique that combines fluoroscopic guidance and ultrasonography to avoid branch vessel injuries [14]. The injury most likely happened when the micropuncture wire was inserted in the right CFA.

## 4. Conclusion

This case underscores the rare but serious vascular complications, such as EPA injury, that can occur even with meticulous technique—particularly in elderly patients with complex vascular anatomy. The proximity of the EPA to the femoral access points makes it vulnerable to inadvertent injury, and haemorrhage from such branches can rapidly become life and limb threatening. Importantly, this case challenges assumptions about the safety of micropuncture techniques and highlights how EPA bleeding can initially mimic benign haematomas. Early recognition of atypical haematoma patterns, prompt imaging and consultation with interventional radiology are critical. We recommend routine fluoroscopic confirmation of the guidewire position within the CFA and consideration of direct ultrasound‐guided wire advancement in selected cases.

## Funding

No funding was received for this manuscript.

## Consent

The patient has provided permission to publish clinical data for her case.

## Conflicts of Interest

The authors declare no conflicts of interest.

## Data Availability

The data that support the findings of this study are available from the corresponding author upon reasonable request.
